# Unveiling the Biogeography and Potential Functions of the Intestinal Digesta- and Mucosa-Associated Microbiome of Donkeys

**DOI:** 10.3389/fmicb.2020.596882

**Published:** 2020-12-04

**Authors:** Ruiyang Zhang, Junpeng Zhang, Wanyi Dang, David M. Irwin, Zhe Wang, Shuyi Zhang

**Affiliations:** ^1^Institute of Equine Sciences, College of Animal Science and Veterinary Medicine, Shenyang Agricultural University, Shenyang, China; ^2^Department of Laboratory Medicine and Pathobiology, University of Toronto, Toronto, ON, Canada

**Keywords:** donkey, 16S rRNA, intestinal microbiota, community structure, metabolic function

## Abstract

The intestinal microbial composition and metabolic functions under normal physiological conditions in the donkey are crucial for health and production performance. However, compared with other animal species, limited information is currently available regarding the intestinal microbiota of donkeys. In the present study, we characterized the biogeography and potential functions of the intestinal digesta- and mucosa-associated microbiota of different segments of the intestine (jejunum, ileum, cecum, and colon) in the donkey, focusing on the differences in the microbial communities between the small and large intestine. Our results show that, Firmicutes and Bacteroidetes dominate in both the digesta- and mucosa-associated microbiota in different intestinal locations of the donkey. Starch-degrading and acid-producing (butyrate and lactate) microbiota, such as *Lactobacillus* and *Sarcina*, were more enriched in the small intestine, while the fiber- and mucin-degrading bacteria, such as *Akkermansia*, were more enriched in the large intestine. Furthermore, metabolic functions in membrane transport and lipid metabolism were more enriched in the small intestine, while functions for energy metabolism, metabolism of cofactors and vitamins, amino acid metabolism were more enriched in the large intestine. In addition, the microbial composition and functions in the digesta-associated microbiota among intestinal locations differed greatly, while the mucosal differences were smaller, suggesting a more stable and consistent role in the different intestinal locations. This study provides us with new information on the microbial differences between the small and large intestines of the donkey and the synergistic effects of the intestinal microbiota with host functions, which may improve our understanding the evolution of the equine digestive system and contribute to the healthy and efficient breeding of donkeys.

## Introduction

Due the similarity of donkey and human milk, the high content of protein and essential amino acids in donkey meat, and the medicinal value of donkey-hide gelatin (Camillo et al., 2018; Xie et al., 2018; Li et al., 2020), animal husbandry of donkeys has occupied an important position in China. To obtain high yields and meet the growing market demand for donkey-related products, donkey breeding in China is attracting increasing attention. However, compared with other livestock, the donkey industry lacks feeding standards and nutritional measures that are suitable for their unique physiology.

Herbivores, such as the donkey, rely on highly dense and diverse microbiota inhabiting their intestine to degrade ingested structural carbohydrates to provide nutrients to support their growth (Sahu and Kamra, 2002; Liu et al., 2014). After millions of years of co-evolution, intestinal microbiota and their hosts have formed complex and mutually adapted micro-ecological systems, with this stable microbiome-host homeostasis being crucial for the up-keep and optimal physiological function of the intestine (Ley et al., 2008). Besides a role in digestion, accumulating evidence indicate that intestinal microbiota are also involved in the efficient utilization of nutrients, digestive tract development, immunity, and other aspects of the health status of the host (Rooks and Garrett, 2016; Malmuthuge and Guan, 2017; Li F. et al., 2019). Thus, understanding the donkey intestinal microbial composition, and its metabolic functions, should help better understand the interactions between the intestinal microbiota and their host and regulation of the donkey’s health status and production performance. However, limited information is currently available regarding donkey intestinal microbial composition and its potential metabolic impact. A few studies have focused on the fecal microbial composition of donkeys (Liu et al., 2014, 2020), and a recent study has investigated the microbial composition and functions of digestive tract digesta in the Dezhou donkey (Liu et al., 2019). To our knowledge, no work has reported on the differences of digesta- and mucosa-associated microbiota in the different intestinal locations in the donkeys.

In the present study, we characterize the digesta- and mucosa-associated microbial community structures and their potential metabolic functions in different segments of the intestine (jejunum, ileum, cecum, and colon). Our findings may contribute to our understanding of microbiome-host homeostasis and interactions in the donkey intestine, and could be used to help develop microbial interventions to improve animal health and production performance.

## Materials and Methods

### Animals and Sample Collection

All animal experiment was conducted in accordance with the requirements of the Experimental Animal Welfare Ethics Committee of Shenyang Agricultural University. All samples in the present study were collected from a breeding farm located in Fuxin City, Liaoning province, China. Digesta and mucosa samples from different intestinal locations of 10 healthy Liaoxi female donkeys (average age of 9 years and body weight of 260 kg) were collected after slaughter. Before slaughter, all donkeys were fed a supplemental diet and roughage three times a day and had free access to water all day. The supplemental diet mainly consisted of corn, soybean meal and wheat bran, and the roughage fraction was cornstalk. Donkeys were fed with 2.5 kg supplemental diet and 6.5 kg roughage a day.

Before slaughter, donkeys had not taken antibiotics for at least 3 months, and were confirmed by the veterinarian as healthy and did not suffer from intestinal diseases during this period. After fasting for approximately 12 h, the selected donkeys for collecting samples were slaughtered. Following slaughter, the organs of the digestive tract were carefully separated and removed, and the digesta and its corresponding mucosal samples from different intestinal locations (jejunum, ileum, cecum, and colon) were collected from each donkey as quickly as possible. A total of 40 digesta and 40 mucosal samples were collected. Upon collection, digesta and mucosa samples were snap-frozen in liquid nitrogen and stored at −80°C until DNA extraction.

### Microbial DNA Extraction, 16S rRNA Sequencing, and Data Processing

Samples of digesta and mucosal tissue from each intestinal location were thawed and fully vortexed, with about 0.3 g of the homogenized sample was used for DNA extraction. After mechanical lysis of the bacterial cells, DNA was isolated using the CTAB-based extraction method described by a previous study (Sun et al., 2008). Extracted DNA was resuspended in Tris-EDTA buffer, and its concentration and quality assessed using a NanoDrop spectrophotometer (Nyxor Biotech, Paris, France).

The V3-V4 region of bacterial 16S rRNA was amplified using the universal primers 338 F (5′-barcode-ACTCCTRCG GGAGGCAGCAG-3′) and 806 R (5′-GGACTACCVGGGTA TCTAAT-3′). PCR products were checked on a 2% agarose gel and target bands were purified using a QIAquick Gel Extraction Kit (Qiagen, Hilden, Germany). A sequencing library was constructed using the TruSeq^®^ DNA PCR-Free Sample Preparation Kit (Illumina, Inc., San Diego, CA, United States), and sequencing was conducted on a Illumina NovaSeq 6000 (Illumina Inc., San Diego, CA, United States).

After truncating the Barcode and primer sequences, FLASH V1.2.7^1^ was used to join the reads of each sample and raw tags were harvested. According to the quality control procedure of Qiime V1.9.1^2^, raw tags were filtered and the clean tags were blasted using the vsearch tool^3^. The software Uparse v7.0.1001^4^ was used to cluster the sequences into operational taxonomic units (OTUs) with a 97% similarity cutoff. The Mothur method was used for species annotation with the SSUrRNA database in SILVA132^5^. Estimates of bacterial community diversity and richness (ACE, Chao 1 and Shannon) were calculated using Qiime V1.9.1, and rarefaction curves (at the level of 3%) were generated to display the overall sequencing information. The 16S rRNA sequencing data from all of the samples used in the present study were deposited into the Sequence Read Archive (SRA) database under accession no. PRJNA656690.

### Predicted Metabolic Functions Based on 16S rRNA Genes

Phylogenetic Investigation of Communities by Reconstruction of Unobserved States (PICRUSt) is a widely used bioinformatics tool for metagenome function prediction based on marker genes, such as 16S rRNA (Langille et al., 2013). In the present study, we used 16S rRNA as a marker gene to predict the metabolic functions of the intestinal microbiota of donkeys. The harvested data on OTUs was standardized by the predicted 16S copy number, and inferred genes and metabolic functions were deduced by blasting the Kyoto Encyclopedia of Genes and Genomes (KEGG) database.

### Statistical Analysis

Microbial data on microbial diversity/richness, different taxonomic levels and microbial functions among the different intestinal locations (longitudinal locations from jejunum to colon) and niches (widthwise locations from lumen to mucosa) were statistically analyzed using IBM SPSS statistics V20.0.0 (IBM Corp., Armonk, NY, United States). Before statistical analysis, the Shapiro-Wilk test was conducted to determine the distribution of the variables. An unpaired Student’s *t*-test or one-way ANOVA was used for analyzing parametric data, and a Mann-Whitney U-test or Kruskal–Wallis one-way analysis of variance (ANOVA) was used for analyzing non-parametric data. Harvested *P*-values were further adjusted with a false discovery rate (FDR) correction. Significant differences were declared at *P* ≤ 0.05.

To evaluate the differences in microbial composition and potential functions in the different intestinal locations and niches, principal coordinate analysis (PCoA) based on the bray-curtis distance and the analysis of molecular variance (AMOVA) were performed using the R program and the software MOTHUR. To explore the correlation between microbiota and its metabolic functions, a correlation analysis was conducted in the present study. The Spearman rank correlation coefficients (r) between bacterial genera and functions were calculated using the *R* program, and correlation coefficients with | r| > 0.5 and *P* < 0.05 were imported into the Gephi v 0.8.2^6^ to visualize networks.

## Results

### Summary of 16S rRNA Amplicon Sequencing Data

To evaluate the impact of the different intestinal locations (longitudinal locations from jejunum to colon) and niches (widthwise locations from lumen to mucosa) on microbial composition and function, a total of 3,435,262, and 3,226,612 high-quality 16S rRNA effective sequences were harvested from 40 digesta and 40 mucosal tissue samples, respectively. On average, 85,882 sequences were obtained per digesta sample and 80,665 sequences per mucosal tissue sample and used for the subsequent analyses. Rarefaction curves were performed at the OTU level, and the results showed that the sampling effort provided sufficient numbers of sequences to measure the majority of the bacterial species (Supplementary Figure 1).

### Overview of Intestinal Microbial Composition and Potential Functions

**FIGURE 1:**
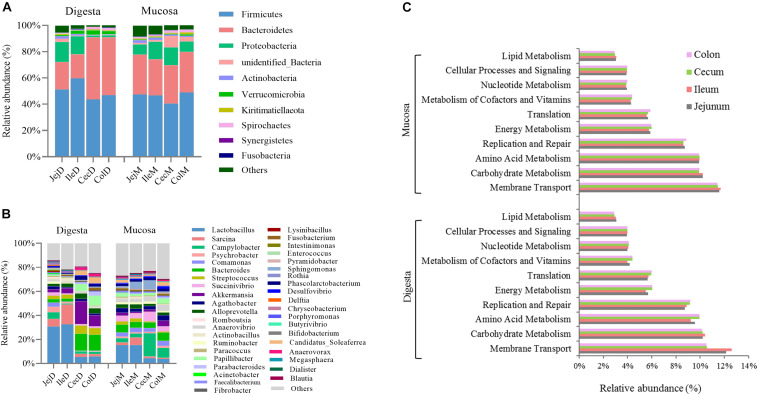
Overview of the intestinal microbial composition and potential functions. Community composition in the digesta- and mucosa-associated microbiota at different intestinal locations at the phylum **(A)** and genus **(B)** levels. **(C)** The top 10 dominant KEGG pathways (expect for poorly characterized bacteria) in both the digesta- and mucosa-associated microbiota at different intestinal locations.

At the phylum level (Figure 1A), except for the digesta-associated microbiota in the large intestine, Firmicutes, Bacteroidetes and Proteobacteria were the three predominant phyla, contributing an average of 83.28∼91.82% of the community in the different intestinal locations (jejunum, ileum, cecum, and colon) and niches (digesta and mucosa). For the digesta-associated microbiota in the large intestine, in addition to Firmicutes and Bacteroidetes, Verrucomicrobia, Spirochetes, and Proteobacteria, but not Proteobacteria, were the predominant phyla. At the genus level (Figure 1B), there were considerable differences in the dominant bacteria in the different intestinal locations and niches. *Lactobacillus*, *Sarcina*, *Akkermansia*, *Campylobacter*, and *Streptococcus* dominated in the digesta samples from the small intestine, and *Akkermansia*, *Bacteroides*, *Papillibacter*, *Streptococcus*, and *Lactobacillus* dominated in the digesta samples from the large intestine; *Lactobacillus*, *Bacteroides*, *Succinivibrio*, *Sarcina*, and *Sphingomonas* dominated in the mucosa samples from the small intestine, and *Campylobacter*, *Succinivibrio*, *Bacteroides*, *Helicobacter*, and *Anaerovibrio* dominated in the mucosa samples from the large intestine. The PCoA profile of the microbiota at the OTU level across all samples based on the Bray-Curtis dissimilarity matrix is presented in Supplementary Figure 2A.

To further explore the potential functions of the intestinal microbiota, gene categories were predicted using PICRUSt. The results (Figure 1C) show that the predicted gene categories belonged to 41 KEGG pathways (level 2), and the top 10 dominated KEGG pathways (expect the poorly characterized bacteria) in the digesta- and mucosa-associated microbiota were involved in membrane transport, carbohydrate metabolism, amino acid metabolism, replication and repair, energy metabolism, translation, metabolism of cofactors and vitamins, nucleotide metabolism, cellular processes and signaling and lipid metabolism. The PCoA profile of microbiota at the KEGG level 2 across all samples based on the Bray-Curtis dissimilarity matrix is presented in Supplementary Figure 2B.

### Community Structure and Potential Functions of the Digesta-Associated Microbiota in the Different Intestinal Locations

**FIGURE 2:**
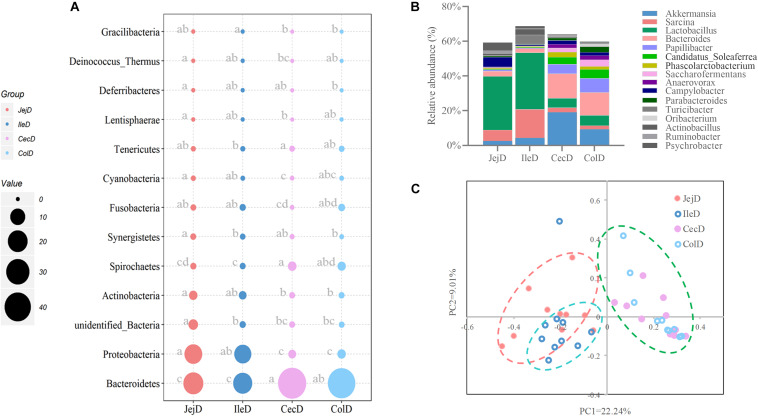
Differences in the community structure of the digesta-associated microbiota at different intestinal locations at the phylum **(A)**, genus **(B)**, and OTU **(C)** levels.

Estimates of microbial diversity (Shannon index) and richness (Chao 1 and ACE) are presented in Supplementary Figure 3, and these results show that the jejunum had a higher (*P* < 0.05) species richness compared to the cecum and colon, as reflected by a higher value of Chao 1. Differences in community structure of digesta-associated microbiota in different intestinal locations at the phylum (Figure 2A), genus (Figure 2B) and OTU (Figure 2C) levels were evaluated. PCoA with Bray–Curtis distances illustrates that the bacterial communities in the different intestinal locations have spatial differences (Figure 2C). An AMOVA analysis confirmed the above result from a statistical point of view (Fs = 3.83329, *P* < 0.001), and the results show that, except for the microbial composition of the cecum and colon (*P* = 0.771), the differences in the microbial composition between the remaining intestinal locations were all significant (*P* < 0.05).

**FIGURE 3:**
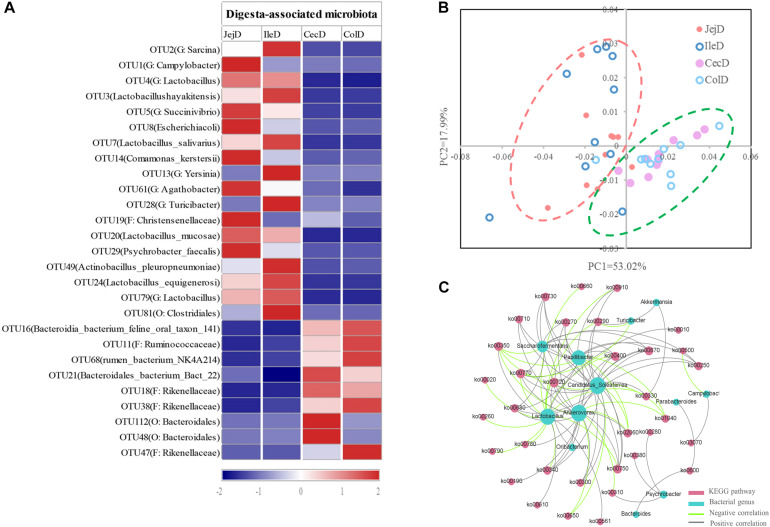
Differences in the dominant OTUs and functions of the digesta-associated microbiota in the different intestinal locations. **(A)** Heatmap of the affected dominant OTUs of the digesta-associated microbiota in the different intestinal locations. **(B)** Principal coordinate analysis (PCoA) profile of the digesta-associated microbial functions (KEGG level 2) in the different intestinal locations. **(C)** Correlation network of abundant (relative abundance >1% in at least in one location) and significantly affected genera and KEGG pathways (level 3). Only correlation coefficients with *P*-value < 0.05 and | r| > 0.5 were used to construct the network.

At the phylum level (Figure 2A), abundance of 12 phyla were significantly different between intestinal locations. Among the dominant phyla, Bacteroidetes were more abundant (*P* < 0.05) in the large intestine, while Proteobacteria were more abundant (*P* < 0.05) in the small intestine. At the genus level (Figure 2B), 16 abundant genera (relative abundance >1% in at least one intestinal location) were significantly different among intestinal locations. In brief, the relative abundance of *Sarcina*, *Lactob*acillus, *Actinobacillus*, *Ruminobacter*, and *Psychrobacter* were higher (*P* < 0.05) in the small intestine, while the relative abundance of *Akkermansia*, *Bacteroides*, *Candidatus_Soleaferrea*, *Papillibacter*, *Saccharofermentans*, *Anaerovorax, Phascolarctobacterium*, *Oribacterium*, and *Parabacteroides* were greater (*P* < 0.05) in the large intestine. At the OTU level (Figure 3A), among the dominant OTUs (relative abundance >1% at least in one location) in digesta-associated microbiota, 27 OTUs were affected by the different intestinal locations. For example, 5 OTUs belonging to genus *Lactob*acillus, including OTU3 (*Lactobacillus hayakitensis*), OTU7 (*Lactobacillus salivarius*), OTU20 (*Lactobacillus mucosae*), OTU24 (*Lactobacillus equigenerosi*), and OTU79 (G: *Lactobacillus*) were more abundant in the digesta-associated microbiota of the small intestine.

**FIGURE 4:**
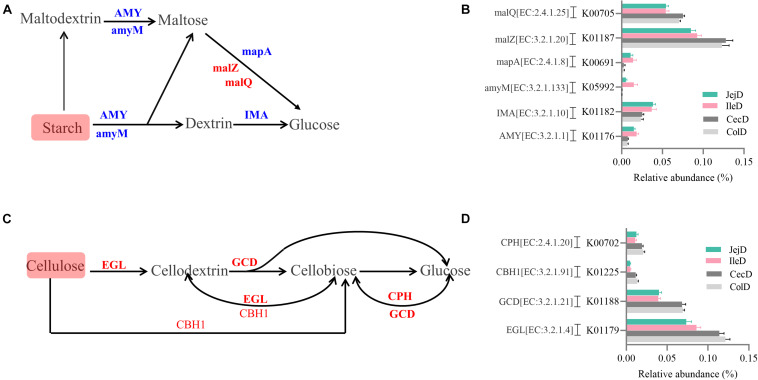
Differences in the metabolic processes of starch and cellulose degradation between the small and large intestines. Overview of the degradation process for starch **(A)** and cellulose **(C)** to glucose. Comparison of the KEGG orthology (KO) genes that differ significantly in abundance in starch **(B)** and cellulose **(D)** degradation between the small and large intestines. Red and blue fonts indicate that the change in abundance of this KO gene was higher or lower, respectively, in the large intestine compared with the small intestine.

The PCoA analysis (Figure 3B) of the microbial functions (KEGG level 2) showed a clear distinction between the samples from the small and large intestines, and the AMOVA analysis confirmed the microbial functions in different intestinal locations had spatial differences (Fs = 3.23438, *P* = 0.026). Of the top 10 dominant KEGG pathways (level 2) in the digesta-associated microbiota (Figure 1C), the relative abundance of energy metabolism, metabolism of cofactors and vitamins, amino acid metabolism in the large intestine were significantly higher (*P* < 0.05) than in the small intestine; whereas the relative abundance of membrane transport and lipid metabolism in the large intestine were significantly lower (*P* < 0.05) than in the small intestine. At the KEGG pathway level 3, we focused on the sub-pathways belonging to the above top 10 pathways (Supplementary Table 1). These results show that 44 sub-pathways were significantly affected (*P* < 0.05) by difference in intestinal location. For example, the affected pathways included valine, leucine, and isoleucine degradation, tyrosine metabolism, tryptophan metabolism and lysine degradation being more enriched (*P* < 0.05) in the small intestine, while other sub-pathways related to amino acids were more enriched in the large intestine. Among the sub-pathways belonging to carbohydrate metabolism, the starch and sucrose metabolism, citrate cycle (TCA) and galactose metabolism were more enriched (*P* < 0.05) in the large intestine, while the butanoate metabolism and propanoate metabolism were more enriched (*P* < 0.05) in the small intestine. For herbivores, the major difference between the small and large intestines is the ability to degrade carbohydrates, as volatile fatty acids (VFA) generated through microbial fermentation is critical to the health and production performance of these animals. Hence, we examined the metabolic processes of starch and cellulose degradation (Figure 4) and VFA formation (Supplementary Figure 4). As for starch degradation (Figures 4A,B), compared with the small intestine, the relative abundance of the KO genes AMY, amyM, IMA, and mapA was decreased (*P* < 0.05), while malZ and malQ increased (*P* < 0.05) in the large intestine. Our results also show that the relative abundance of KEGG orthology (KO) genes involved in cellulose degradation were all higher (*P* < 0.05) in the large intestine when compared to the small intestine (Figures 4C,D). Among the 25 affected KO genes involved in VFA formation (Supplementary Figure 4), the relative abundance of 6 KO genes was decreased (*P* < 0.05), while the other 19 KO genes increased (*P* < 0.05) in the large intestine compared with the small intestine.

To further explore the relationship between intestinal microbiota and their potential microbial functions, we used the abundant (relative abundance >1% in at least in one location) and significantly affected microbial genera and KEGG pathways (level 3) to construct a Spearman’s correlation network (Figure 3C). The results of this network analysis showed that there were complex connections between the microbial genera and their functions. The microbial genera *Anaerovorax*, *Lactobacillus*, *Candidatus_Soleaferrea*, *Papillibacter*, and *Saccharofermentans* had the most connections with the KEGG pathways. For example, *Lactobacillus* was positively correlated (*P* < 0.05) with phosphotransferase system (ko02060), tyrosine metabolism (ko00350), fatty acid biosynthesis (ko00610), glycerolipid metabolism (ko00561), and glycolysis/gluconeogenesis (ko00010), while being negatively correlated (*P* < 0.05) with glycine, serine and threonine metabolism (ko00260), arginine and proline metabolism (ko00330), histidine metabolism (ko00340), and citrate cycle (ko00020). In addition, valine, leucine and isoleucine biosynthesis (ko00290) was positively correlated (*P* < 0.05) with *Akkermansia*, *Candidatus Soleaferrea*, and *Psychrobacter*, while negatively correlated with *Lactobacillus* and *Turicibacter*.

### Community Structure and Potential Functions of the Mucosa-Associated Microbiota in the Different Intestinal Locations

**FIGURE 5:**
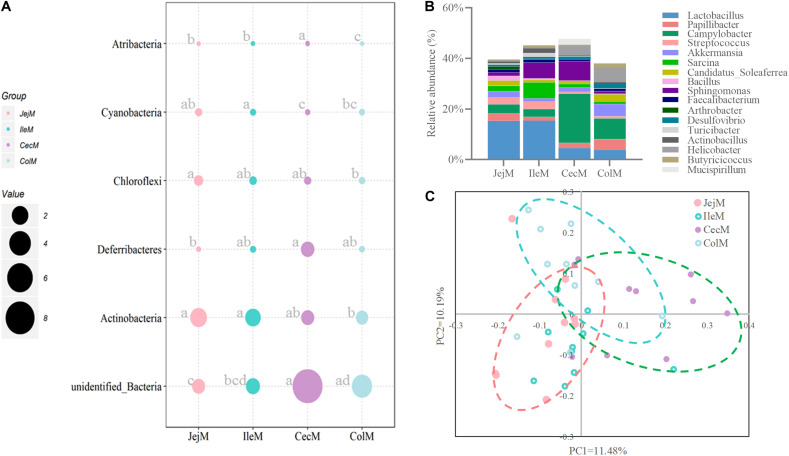
Differences in the community structure of the mucosa-associated microbiota in the different intestinal locations at the phylum **(A)**, genus **(B)**, and OTU **(C)** levels.

As for richness and diversity of mucosa-associated microbiota (Supplementary Figure 3), the results show that the Shannon index, Chao 1 and ACE of the microbiota of the jejunum is significantly higher (*P* < 0.05) than in the cecum or colon. Differences in community structure of mucosa-associated microbiota in different intestinal locations at the phylum (Figure 5A), genus (Figure 5B) and OTU (Figure 5C) levels were evaluated. The PCoA with Bray–Curtis distances (Figure 5C) revealed that the mucosa-associated microbiota in the small intestine or the large intestine clustered more closely together than between the small and large intestines. The AMOVA analysis suggested that there were significant differences (Fs = 1.92523, *P* < 0.001) in mucosa-associated microbial composition at the different intestinal locations (except for ileum vs. jejunum, *P* = 0.190).

**FIGURE 6:**
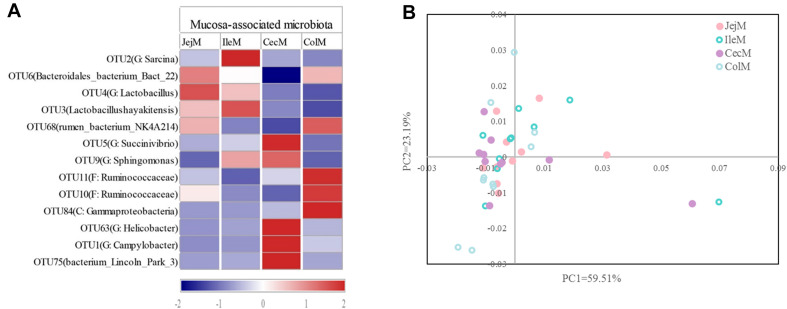
Differences in the dominant OTUs and functions of the mucosa-associated microbiota in different intestinal locations. **(A)** Heatmap of the affected dominant OTUs of the mucosa-associated microbiota in the different intestinal locations. **(B)** Principal coordinate analysis (PCoA) profile of the mucosa-associated microbial functions (KEGG level 2) in the different intestinal locations.

At the phylum level (Figure 5A), the predominant phyla Firmicutes, Bacteroidetes and Proteobacteria in mucosa-associated microbiota were not affected by intestinal location. However, the relative abundance of Cyanobacteria, Actinobacteria, and Chloroflexi were higher (*P* < 0.05), and Deferribacteres and Atribacteria were lower (*P* < 0.05) in the small intestine compared with the large intestine. At the genus level (Figure 5B), 17 abundant genera (relative abundance >1% in at least one intestinal location) of mucosa-associated microbiota displayed significant differences in abundance across intestinal locations. Briefly, the relative abundance of *Lactobacillus*, *Streptococcus*, *Sarcina*, *Faecalibacterium*, *Actinobacillus*, and *Arthrobacter* were greater in the mucosa-associated microbiota of the small intestine, while the relative abundance of *Papillibacter*, *Campylobacter*, *Akkermansia*, *Candidatus_Soleaferrea*, *Desulfovibrio*, *Helicobacter*, *Mucispirillum*, and *Butyricicoccus* were greater in the mucosa-associated microbiota of the large intestine. At the OTU level (Figure 6A), 13 dominant OTUs (relative abundance >1% in at least one location) in the mucosa-associated microbiota were affected by intestinal location. For example, our results showed that OTU6 (*Bacteroidales bacterium Bact 22*), OTU4 (G: *Lactobacillus*) and OTU3 (*Lactobacillus hayakitensis*) were more abundant in the mucosa-associated microbiota of the small intestine, while OTU5 (G: *Succinivibrio*), OTU11 (F: Ruminococcaceae), OTU10 (F: Ruminococcaceae), OTU84 (C: Gammaproteobacteria), OTU63 (G: *Helicobacter*), OTU1 (G: *Campylobacter*), and OTU75 (*bacterium Lincoln Park 3*) were more abundant in the mucosa-associated microbiota of the large intestine.

**FIGURE 7:**
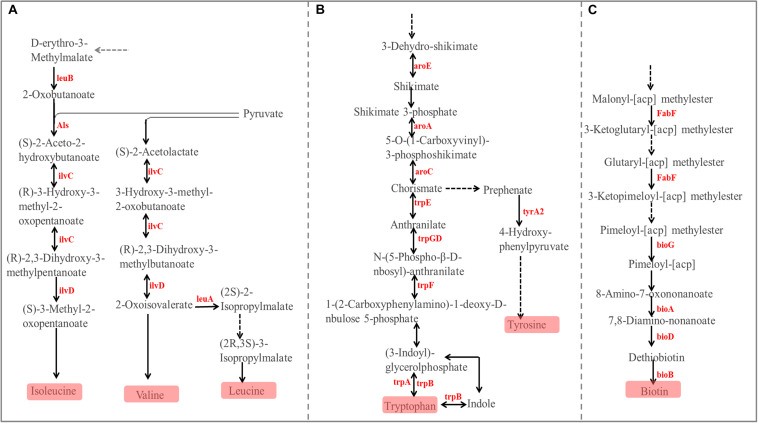
Differences in the metabolic pathway for Phenylalanine, tyrosine, and tryptophan biosynthesis **(A)**, Valine, leucine, and isoleucine biosynthesis **(B)**, and Biotin metabolism **(C)** between the mucosa-associated microbiota in the small and large intestines. Red font indicates that the abundance of this KO genes is higher in the large intestine compared with the small intestine.

The PCoA (Figure 6B) and AMOVA analysis of the mucosa-associated microbial functions (KEGG level 2) showed that there were no differences between the samples from the small and large intestines (Fs = 0.138522, *P* = 0.98). For further analysis, we carried out a statistical analysis of the detailed functional categories of the mucosa-associated microbiota. Of the top 10 dominant KEGG pathways (level 2) in the mucosa-associated microbiota (Figure 1C), the small intestine had a higher (*P* < 0.05) relative abundance of carbohydrate metabolism pathways than the large intestine, while other dominant KEGG pathways were not affected (*P* > 0.05) by intestinal location. At the KEGG level 3 (Supplementary Table 2), among the sub-pathways belonging to the top 10 dominant KEGG pathways, the relative abundance of phenylalanine, tyrosine and tryptophan biosynthesis, valine, leucine and isoleucine biosynthesis, C5-Branched dibasic acid metabolism, biotin metabolism and pantothenate and CoA biosynthesis were lower (*P* < 0.05), while the relative abundance of tyrosine metabolism, glycolysis/gluconeogenesis and phosphotransferase system (PTS) were higher (*P* < 0.05) in the mucosa-associated microbiota of the small intestine compared with the large intestine. The metabolic routes of phenylalanine, tyrosine and tryptophan biosynthesis, valine, leucine and isoleucine biosynthesis, and biotin metabolism are summarized in Figure 7 and Supplementary Figure 5. Our results show that the mucosa-associated microbiota resident in the large intestine tend to synthetize branched-chain and aromatic amino acids and biotin.

To further explore the relationship between mucosa-associated microbiota and potential microbial functions, we conducted a correlation analysis between the abundant (relative abundance >1% in at least in one location) and significantly affected microbial genera and KEGG pathways (level 3). Our results (Supplementary Table 3) show that there were fewer connections between the bacterial genera and functions of the mucosa-associated microbiota than those of digesta-associated microbiota. Only 5 genera had connections with KEGG pathways in the mucosa-associated microbiota, for example, *Lactobacillus* and *Streptococcus* were positively correlated (*P* < 0.05) with glycolysis/gluconeogenesis and phosphotransferase system (PTS), while they were negatively correlated (*P* < 0.05) with valine, leucine and isoleucine biosynthesis. *Sphingomonas* was positively correlated (*P* < 0.05) with tyrosine metabolism and valine, leucine and isoleucine biosynthesis, while it was negatively correlated (*P* < 0.05) with pantothenate and CoA biosynthesis and phenylalanine, tyrosine and tryptophan biosynthesis.

### Comparison of the Community Structure and Potential Functions Between Digesta- and Mucosa-Associated Microbiota From Different Intestinal Locations

When comparing the diversity and richness between the digesta- and mucosa-associated microbiota in the different intestinal locations (Supplementary Figure 6), except for the Shannon index for the cecum, the Shannon index, Chao 1 and ACE were all higher (*P* < 0.05) in the mucosa samples than in the digesta samples. At the phylum level (Supplementary Figure 7A), among three dominant phyla (Firmicutes, Bacteroidetes and Proteobacteria), the relative abundance of Bacteroidetes was significantly higher (*P* < 0.05) in the mucosa samples of the small intestine, while it was lower (*P* < 0.05) in the mucosa samples of the large intestine than in their corresponding digesta samples. The relative abundance of Proteobacteria was significantly higher (*P* < 0.05) in the digesta samples from the jejunum, while it was lower (*P* < 0.05) in the digesta samples of the large intestine than those from its corresponding mucosa samples.

At the genus level, we compared the abundant (relative abundance >1% in at least one niche) genera between the digesta- and mucosa-associated microbiota in different intestinal locations. Our results (Supplementary Figure 7A) show that, the relative abundance of *Lactobacillus* and *Comamonas* were higher, while *Bacteroides*, *Campylobacter*, *Succinivibrio*, *Papillibacter*, and *Alloprevotella* were lower (*P* < 0.05) in the digesta samples compared with their corresponding mucosa samples from the small intestine. The relative abundance of *Akkermansia*, *Streptococcus*, *Alloprevotella*, *Candidatus Soleaferrea*, and *Ruminobacter* were higher (*P* < 0.05), while *Campylobacter*, *Succinivibrio*, *Agathobacter*, *Anaerovibrio*, and *Comamonas* were lower (*P* < 0.05) in the digesta samples when with their corresponding mucosa samples from the large intestine.

As for microbial functions (Supplementary Figure 7B), in the small intestine, the mucosa-associated microbiota had a higher (*P* < 0.05) activity for amino acid metabolism, energy metabolism, metabolism of cofactors and vitamins, cell motility and transcription, and a lower activity for infectious diseases compared with the digesta-associated microbiota. However, in the large intestine, the mucosa-associated microbiota had a higher (*P* < 0.05) activity in membrane transport, cell motility, xenobiotics biodegradation and metabolism, signal transduction and environmental adaptation, and a lower (*P* < 0.05) activity for replication and repair, nucleotide metabolism, glycan biosynthesis and metabolism, enzyme families and transport and catabolism compared with the digesta-associated microbiota.

## Discussion

Due to the scarcity of studies on the intestinal microbiota of donkeys, the present study aimed to examine the biogeography and potential functions of digesta- and mucosa-associated microbiota in different location of the intestine. Results from both the PCoA and the AMOVA analyses confirmed that significant differences in both digesta- and mucosa-associated microbiota exist among different intestinal locations. The results showed that the diversity and/or richness of digesta- and mucosa-associated microbiota in the large intestine were higher than those in the small intestine. This may be due to the differences of available oxygen, types of nutrients and the internal environment in the small and large intestine. At the phylum level, Firmicutes and Bacteroidetes dominated in both the digesta and mucosa samples of different intestinal locations in the donkey; Bacteroidetes was significantly enriched in the digesta-associated microbiota of the large intestine, and these two dominant phyla were not affected by intestinal location in the mucosa-associated microbiota. Previous studies showed that Firmicutes and Bacteroidetes were the most two dominant phyla in several other herbivorous animals, such as the goat (Li B. et al., 2019), cattle (Mao et al., 2015), and horse (Al Jassim and Andrews, 2009), which may relate to their dietary characteristics and the necessity of digesting large amounts of fibrous carbohydrate. The phylum Bacteroidetes are known to possess abundant genes that encode carbohydrate active enzymes and are able to switch readily between different types of energy source in the gut depending on availability (Flint et al., 2012). Hence, enrichment of Bacteroidetes in the large intestine may help the host adapt to the complex internal environment and degradation of non-fibrous carbohydrate in the large intestine.

At the genus level, the dominant genera in the digesta- and mucosa-associated microbiota in the different intestinal locations exhibited huge differences. For the digesta-associated microbiota, *Lactobacillus*, *Sarcina*, and *Akkermansia* dominated in the small intestine, while *Akkermansia*, *Bacteroides*, and *Papillibacter* dominated in the large intestine; for the mucosa-associated microbiota, *Lactobacillus*, *Bacteroides*, and *Succinivibrio* dominated in the small intestine, while *Campylobacter*, *Succinivibrio*, and *Bacteroides* dominated in the large intestine. Results of a statistical analysis showed that *Lactobacillus* and *Sarcina* were significantly enriched in the small intestine, and *Akkermansia*, *Bacteroides*, and *Papillibacter* were significantly enriched in the large intestine. Except for *Bacteroides*, the same result concerning the affected genera was also found in the mucosa-associated microbiota of the small and large intestine. This result suggests that the microbiota that colonized the mucosa originated from the digesta in the intestinal lumen. The dominant position of *Lactobacillus* at the genus level in the small intestine of the donkey was consistent with the results of a previous study (Liu et al., 2019). *Lactobacillus* was reported to have non-fibrous carbohydrate-degrading (e.g., pentoses, hexoses, and starch) capacity (Garrity, 2005), be involved the deconjugation of bile salts (Bao et al., 2012), and inhibit the proliferation of pathogenic bacteria by producing antimicrobial substances (such as bacteriocins and lactate) or competing with pathogenic bacteria for mucosal adhesion sites and nutrients (Savadogo et al., 2006; Umu et al., 2017). The metabolic characteristics of *Lactobacillus* correspond to the high relative abundance of lipid metabolism (and also its sub-pathway fatty acid metabolism) and glycolysis/gluconeogenesis found in the small intestine. *Sarcina*, a member of Clostridiaceae, was implicated in the inflammatory process, which may be related to the production of butyrate by its sugar-fermenting capacities (Canale-Parola, 1970; Getachew et al., 2018). In addition, results of a single-cell transcriptome analysis in humans showed that the small intestine may have a strong defense response to bacterial infection (Wang et al., 2020), and hence, the relatively high abundance of *Lactobacillus* and *Sarcina* in the digesta- and mucosa-associated microbiota might contribute to a higher immune resistance of the small intestine. A previous study showed a highest species distribution proportion observed for *Akkermansia* in the fecal microbiota of donkeys (Liu et al., 2014). Consistent with this reported result, our results also indicate abundant *Akkermansia* colonized in the digesta and mucosa of the large intestine of the donkey. Available evidence from human and animal studies confirm that *Akkermansia* (belonging to the phylum Verrucomicrobia) is a mucin-degrading bacterial species which is able to utilize mucus glycans as the only constant source of carbon and nitrogen (Zhou, 2017). As the result of mucus degradation, the metabolites such as oligosaccharides and VFA produced by *Akkermansia* can then promote cross-talk between the intestinal microbiota and the host, and hence, maintain intestinal health (Belzer and De Vos, 2012). Meanwhile, according to our results that there were a large number of intestinal probiotics (*Lactobacillus* and *Akkermansia*) colonized in donkey intestine, we speculated that these probiotics could be manipulated in donkey to exert its probiotic effects, such as regulating the intestinal microbial balance, maintaining intestinal immunity and improving its production performance (Suda et al., 2014; Zhao et al., 2019). Acting as a prevalent hindgut symbiont in humans and other animals, many species of the genus *Bacteroides* contribute to the digestion of a variety of plant polysaccharide, including fibrous substances (Henderson and Demeyer, 1989; Qin et al., 2010; Zhang et al., 2018). Besides its activity at degradation, *Bacteroides* also plays a crucially important role in shaping epithelial immunity and maintaining the intestinal microecological balance (Hooper, 2004; Zhang et al., 2018).

Our results also found that *Ruminobacter* was enriched in the digesta-associated microbiota, and that *Streptococcus* and *Faecalibacterium* were enriched in the mucosa-associated microbiota resident in the small intestine. *Ruminobacter*, belongs to the family Succinivibrionaceae, which was reported to possess amylase activity and ferment starch into succinate, acetate, and formate (Harlow et al., 2016). The amylolytic bacteria – *Streptococcus* are always found in the rumen of ruminants fed high-concentrate diets (Khafipour et al., 2016). Similar to the genus *Lactobacillus*, its fermentation product lactate makes the intestine mucosa an acidic environment, hence, *Lactobacillus* and *Streptococcus* were both considered as putative beneficial probiotics for horses (Islam and Lee, 2018; Schoster, 2018). As the butyrate-producing bacteria *Faecalibacterium* was found to colonize the small intestinal mucosa in the present study, its fermentation end product butyrate could play a role in promoting mucosal proliferation and differentiation (Li B. et al., 2019). In addition, we also observed that *Saccharofermentans* and *Phascolarctobacterium* were enriched in the digesta-associated microbiota resident in the large intestine. *Saccharofermentans*, a member of Clostridiales, is a cellulose-degrading bacteria that is able to secret cellulosomes and provide fermentation substrates for acidogenic bacteria (Perea et al., 2017; Han et al., 2020). The Clostridia genus *Phascolarctobacterium* acts as fiber fermenter, and can utilize succinate generated by other bacteria to produce acetate and propionate (Connors et al., 2019; Kauter et al., 2019). Overall, the above results show that the digesta- and mucosa-associated microbiota resident in the different intestinal locations coordinate with intestine functions, and are especially closely related to the available fermentation substrates. These results provide effective information about the normal intestinal microbial structure of donkeys, which contribute to the subsequent development of donkey-specific microbial control measures.

To explore the metabolic functions of the intestine microbiota, we used PICRUSt to determine the potential functions of digesta- and mucosa-associated microbiota in the different intestinal locations. Our results revealed that the major functions of both the digesta- and mucosa-associated microbiota in the different intestinal locations were membrane transport, carbohydrate metabolism, amino acid metabolism, replication and repair and energy metabolism, which is consistent with the functions of the intestinal microbiota in other animals (Mao et al., 2015; Li B. et al., 2019). This result corresponds to the basal metabolic functions of microbiota and the nutrients (such as carbon and nitrogen sources) required for their growth and proliferation (Lamendella et al., 2011). Our results indicate that there are huge differences in the functions of the digesta-associated microbiota between the small and large intestines, as indicated by the results of the PCoA and AMOVA analyses. Through statistical analysis, we found that membrane transport and lipid metabolism were more enriched in the digesta-associated microbiota of the small intestine, while energy metabolism, metabolism of cofactors and vitamins, and amino acid metabolism were more enriched in the large intestine. This result suggest that the microbial metabolic activities in the large intestine are more active than in the small intestine. The digestion of lipids in mammals is mainly completed in the small intestine, and intestinal microbiota participates in lipid metabolism (Caesar et al., 2010; Joyce et al., 2014), and correspondingly, our results show that lipid metabolism was enriched in the small intestine. The enrichment of amino acid metabolism (the affected sub-pathways include both the metabolism and biosynthesis) in the large intestine is consistent with the increased expression of transporter proteins and digestive enzymes for amino acids, in the large intestine compared to the small intestine, thus, amino acid metabolism in the small intestine is more dependent on the host and in the large intestine is more dependent on the microbiota (Bröer, 2008; Wang et al., 2020); on the other hand, the lower levels of carbon sources reaching the large intestine forces the microorganisms to utilize greater amounts of nitrogen sources and thus, catabolize undigested proteins and amino acids that originated from the small intestine (Hendriks et al., 2012; Sun et al., 2015). For herbivores, the amounts and types of carbohydrates flowing into the small and large intestines are different, as well as the fermentation capacity of microbiota (Gidenne and Perez, 1993; Gilbert et al., 2015). Based on this difference, we focused on the routes for starch and fiber degradation and VFA formation. Correspondingly, our results show that the degradation of starch by the microbiota that colonize the large intestine is weakened, and the ability to degrade cellulose and generate VFA is enhanced compared to the small intestine, as indicated by the decreased number of KO genes involved in starch degradation and the increased number of KO genes in cellulose degradation and VFA formation. Hence, the above results show that through co-evolution the intestinal microbiota have formed stable functions that are synergistic with the host functions. The microbiota resident in the small intestine play an important role in the digestion of starch and lipids, while the microbiota that colonize the large intestine focus more on the degradation of fiber and amino acids.

For the functions of the mucosa-associated microbiota, there were only minor differences in the different intestinal locations. At KEGG level 2, only carbohydrate metabolism was affected by intestinal location and was enriched in mucosa-associated microbiota of the small intestine. At KEGG level 3, among the sub-pathways belonging to the dominat KEGG pathways, only 8 sub-pathways in the mucosa-associated microbiota were affected, and these sub-pathways in digesta-associated microbiota were also affected by intestinal location. Our results show that tyrosine metabolism, glycolysis/gluconeogenesis and phosphotransferase system were enriched in the small intestine, while phenylalanine, tyrosine and tryptophan biosynthesis, valine, leucine and isoleucine biosynthesis, C5-Branched dibasic acid metabolism, biotin metabolism and pantothenate and CoA biosynthesis were enriched in the large intestine. The enrichment of glycolysis/gluconeogenesis in the digesta- and mucosa-associated microbiota in the small intestine may once again reveal that the small intestine has a strong ability to digest starch, with the microbiota then using the monosaccharides produced by the degradation of starch to synthesize lactate. By screening KO genes involved in the affected metabolic sub-pathways involved in amino acids, our results show that the microbiota in the large intestine appeared to be more inclined to synthesize branched-chain and aromatic amino acids. Besides the catabolism of amino acids by the microbiota resident in the lumen, the large intestine may also have abilities in amino acid uptake, as indicated by the high expression of some amino acid transporters, such as L-amino acid transporter 1 which conveys the transport of branched-chain and aromatic amino acids (Blachier et al., 2007; Hendriks et al., 2012). In addition, the synthetic amino acids may be partly used to support the rapid turnover of the large intestinal epithelium (Backes et al., 2002; Blachier et al., 2007). Previous studies reported that the feedback inhibition of enzymes such as acetohydroxyacid synthase and isopropylmalate synthetase by branched-chain amino acids, and chorismate mutase and prephenate dehydratase by aromatic amino acid played an important role in their biosynthesis pathways (Ikeda and Katsumata, 1992; Park and Lee, 2010). Hence, the above results reveal that the synthesis of these amino acids can also be self-regulated through feedback inhibition and undergoes dynamic changes with the host status. Biotin is an essential cofactor for many central metabolic reactions (such as fatty acid metabolism and amino acid metabolism), and is important for mucosa immunity (Zoetendal et al., 2012; Lakhan and Said, 2017). The enrichment of biotin metabolism in the mucosa-associated microbiota resident in the large intestine may contribute to the complex metabolic processes and immune maintenance in the large intestine.

## Conclusion

In conclusion, the present study unveiled the biogeography and potential functions of the intestinal digesta- and mucosa-associated microbiota of the donkey. Our results demonstrate that the microbial community structure and its potential functions are synergistic with host functions. Overall, the starch-degrading and acid-producing (butyrate and lactate) microbiota were more enriched in the small intestine, while fiber-degrading and mucin-degrading bacteria were more enriched in the large intestine. The microbiota resident in the small intestine were more inclined to have functions such as membrane transport and lipid metabolism, while microbiota resident in the large intestine were more inclined to have functions such as energy metabolism, metabolism of cofactors and vitamins, amino acid metabolism. The microbial composition and functions among intestinal locations differed greatly, while the mucosal differences were smaller. These findings gain more insight into intestinal microbiome in donkeys, and future studies should be combined with host intestinal functions (transcriptome or single cell sequencing, etc.) of the donkey to confirm the above-mentioned synergistic effects.

## Data Availability Statement

The 16S rRNA sequencing data from all of the samples used in the present study were deposited into the Sequence Read Archive (SRA) database under accession no. PRJNA656690.

## Ethics Statement

The animal study was reviewed and approved by the Experimental Animal Welfare Ethics Committee of Shenyang Agricultural University.

## Author Contributions

SZ and ZW designed the study. RZ, JZ, and WD collected the samples. RZ analyzed the data and wrote the manuscript. DI, ZW, and SZ revised the manuscript. All authors contributed to the article and approved the submitted version.

## Conflict of Interest

The authors declare that the research was conducted in the absence of any commercial or financial relationships that could be construed as a potential conflict of interest.

## Footnotes

1 http://ccb.jhu.edu/software/FLASH/

2 http://qiime.org/scripts/split_libraries_fastq.html

3 https://github.com/torognes/vsearch

4 http://www.drive5.com/uparse/

5 http://www.arb-silva.de/

6 https://gephi.org/
