# Supplementary material for: Unveiling the Biogeography and Potential Functions of the Intestinal Digesta- and Mucosa-Associated Microbiome of Donkeys
Source: Front Microbiol. 2020 Dec 4;11:596882. doi: 10.3389/fmicb.2020.596882 (PMC7793809; doi:10.3389/fmicb.2020.596882)
Supplement: Supplementary file 1 [file Data_Sheet_1.pdf]

Supplementary Material

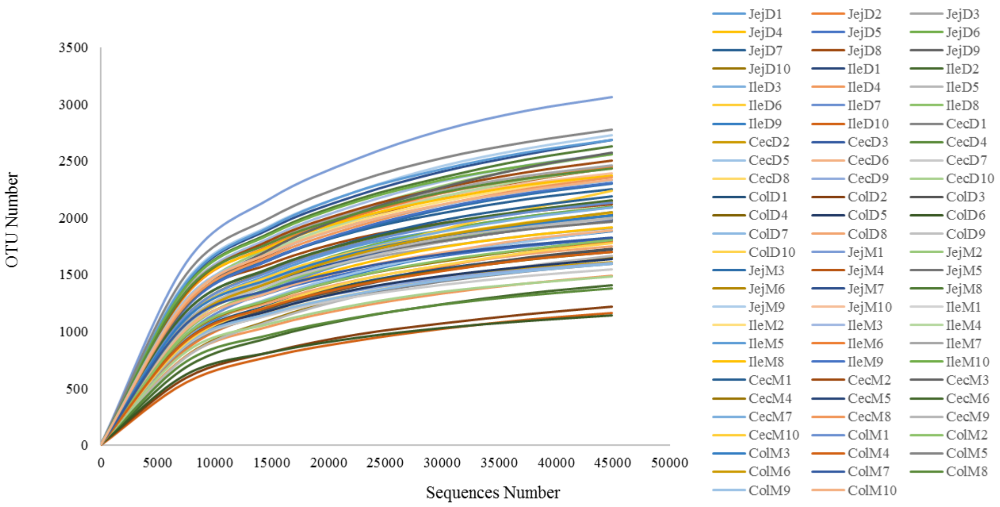

Figure S1 Summary of the rarefaction results based on the operational taxonomic units (OTU) at 3% divergence for each sample. JejD, IleD, CecD and ColD represent the digesta samples from the jejunum, ileum, cecum and colon, respectively; JejM, IleM, CecM and ColM represent the mucosa samples from the jejunum, ileum, cecum and colon, respectively.

A

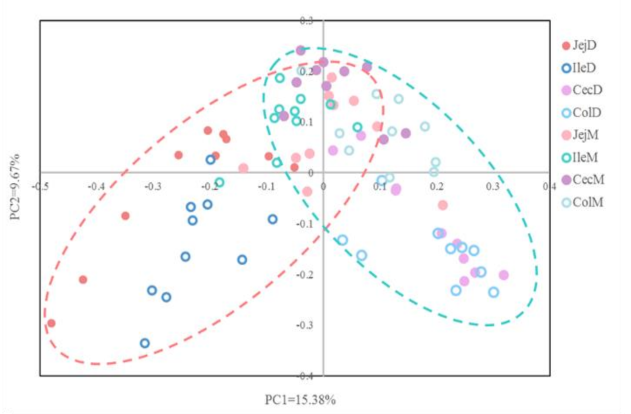

B

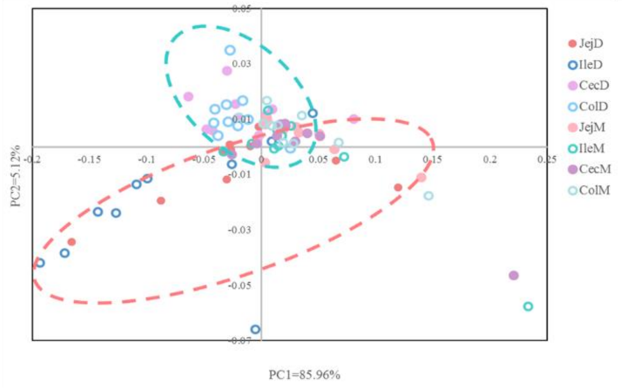

Figure S2 Principal coordinate analysis (PCoA) profiles of microbiota (OTU level; A) and their functions (KEGG level 2; B) across all samples based on the Bray-Curtis dissimilarity matrix. JejD, IleD, CecD and ColD represent the digesta samples from the jejunum, ileum, cecum and colon, respectively; JejM, IleM, CecM and ColM represent the mucosa samples from the jejunum, ileum, cecum and colon, respectively.

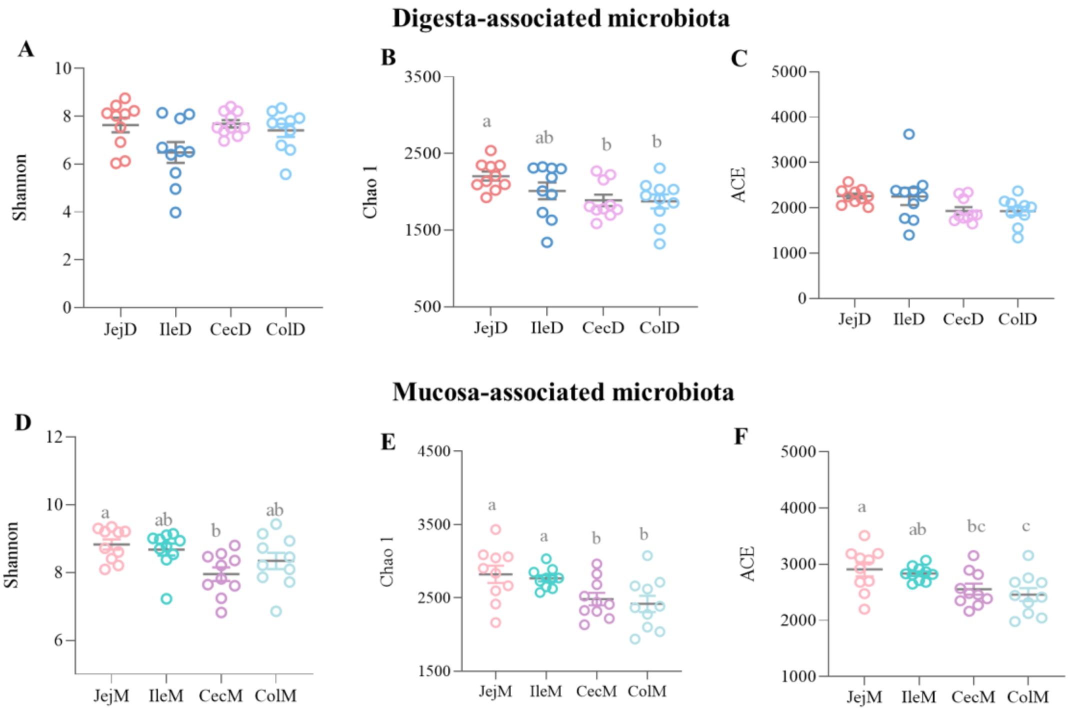

Figure S3 Summary overview of the diversity and richness of digesta- and mucosa-associated microbiota in different intestinal locations. JejD, IleD, CecD and ColD represent the digesta samples from the jejunum, ileum, cecum and colon, respectively; JejM, IleM, CecM and ColM represent the mucosa samples from the jejunum, ileum, cecum and colon, respectively.

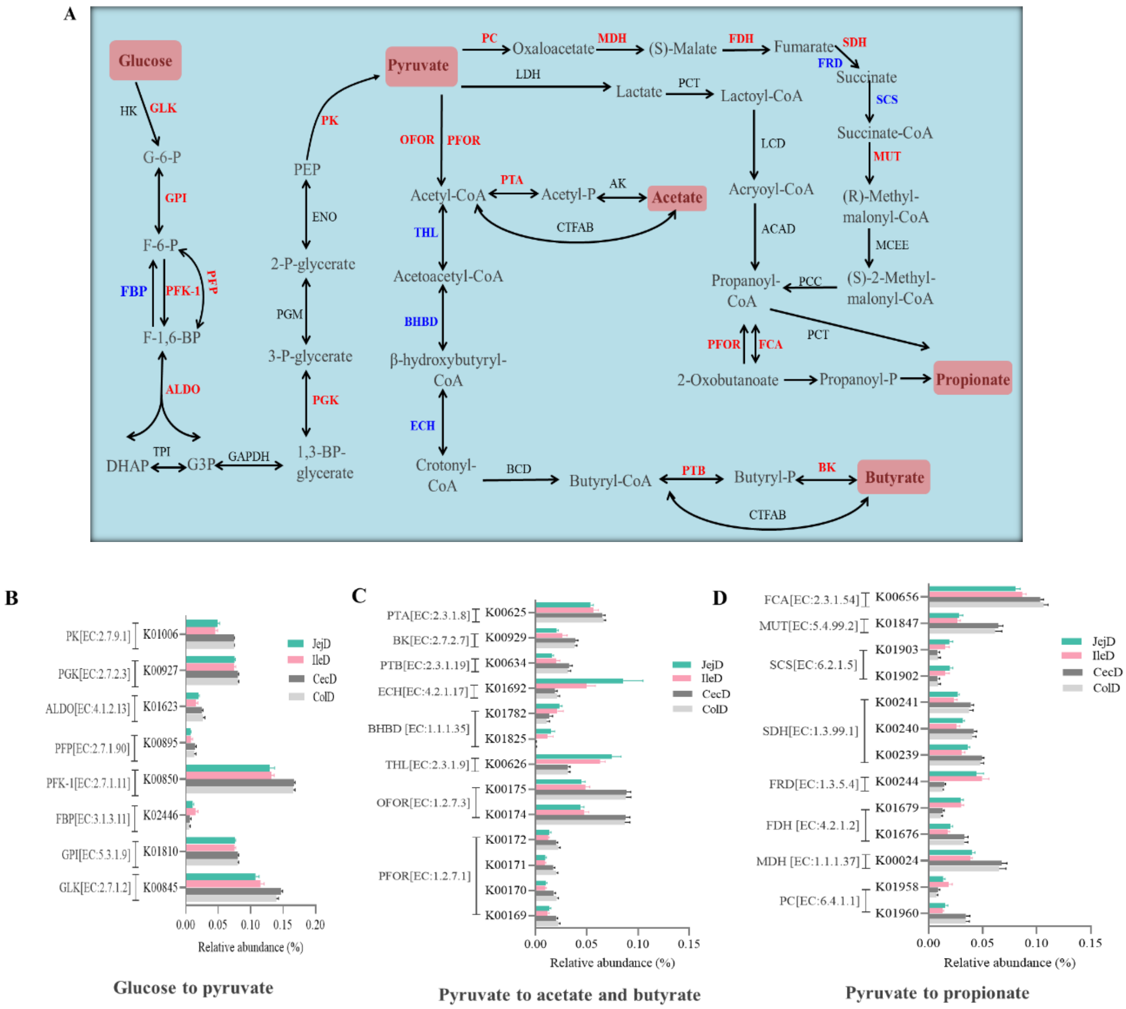

Figure S4 Differences in the metabolic processes of volatile fatty acids (VFA) formation between the small and large intestines. (A) Overview of VFA formation from glucose to acetate, propionate and butyrate. Red and blue fonts indicate that the abundance of this KO gene was higher or lower, respectively, in the large intestine compared with small intestine. (B) Comparison of the KEGG orthology (KO) genes that significantly differed in the VFA formation process between the small and large intestines.

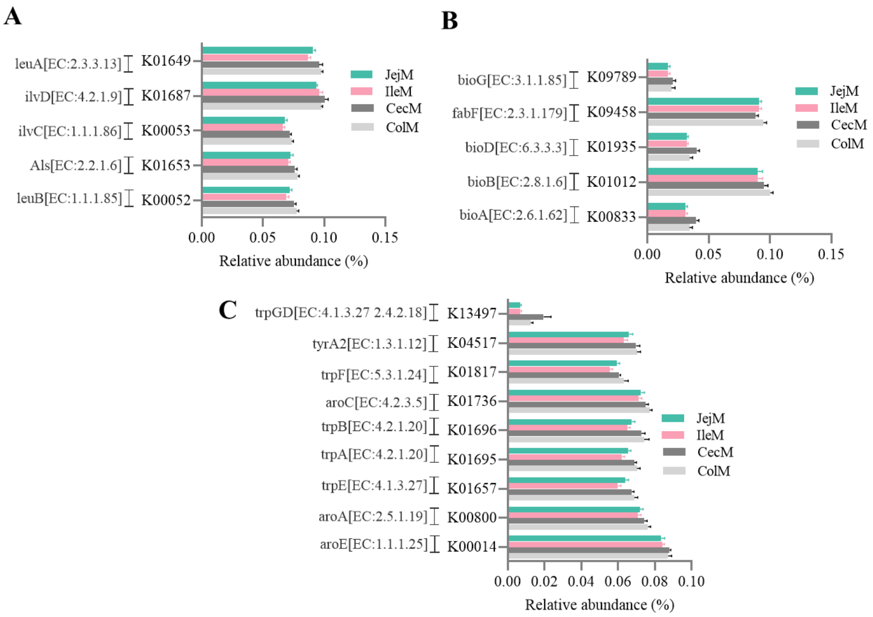

Figure S5 Differences in the KEGG orthology (KO) genes related to the metabolic pathways for Phenylalanine, tyrosine and tryptophan biosynthesis (A), Valine, leucine and isoleucine biosynthesis (B), and Biotin metabolism (B) between the small and large intestines.

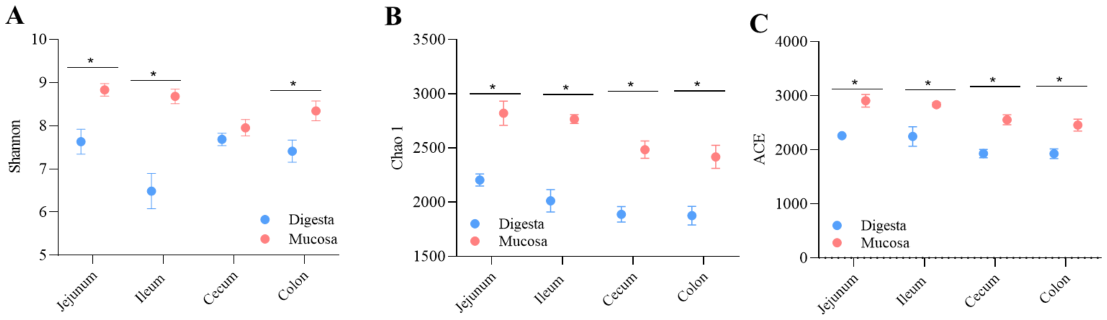

Figure S6 Comparison of the microbial diversity (A) and richness (B and C) between the digesta- and mucosa-associated microbiota at different intestinal locations.

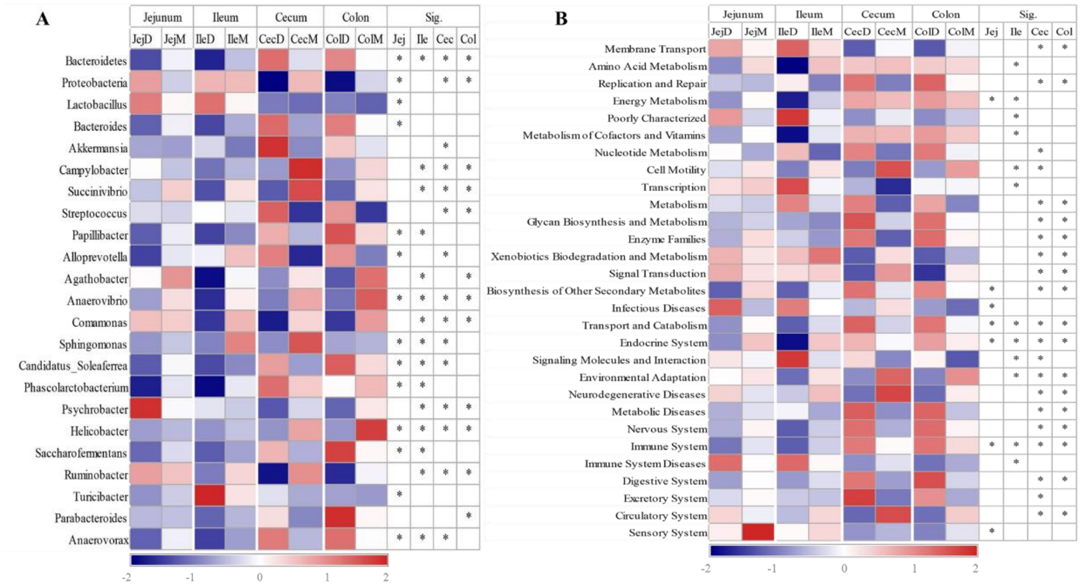

Figure S7 Comparison of the microbial composition (at the phylum and genus level; A) and functions (level 2; B) between the digesta- and mucosa-associated microbiota at different intestinal locations.
