# Supplementary material for: Unveiling the Biogeography and Potential Functions of the Intestinal Digesta- and Mucosa-Associated Microbiome of Donkeys
Source: Front Microbiol. 2020 Dec 4;11:596882. doi: 10.3389/fmicb.2020.596882 (PMC7793809; doi:10.3389/fmicb.2020.596882)
Supplement: Supplementary file 2 [file Data_Sheet_2.pdf]

Supplementary Material

Table S1 Affected sub-pathways belonging to the top 10 pathways at KEGG level 2 in the digesta-associated microbiota. Numbers in the same row with different superscripts indicate a significant difference ($P < 0.05$).

KEGG_Pathways	Jejunum	Ileum	Caecum	Colon	SEM	*P* value
**Amino Acid Metabolism**						
Amino acid related enzymes	1.43^{b}	1.43^{b}	1.52^{a}	1.53^{a}	0.011	<0.001
Arginine and proline metabolism	1.13^{b}	1.09^{b}	1.26^{a}	1.25^{a}	0.015	<0.001
Alanine, aspartate and glutamate metabolism	1.00^{b}	0.97^{b}	1.09^{a}	1.1^{a}	0.012	<0.001
Cysteine and methionine metabolism	0.91^{c}	0.93^{bc}	0.96^{ab}	0.97^{a}	0.006	<0.001
Phenylalanine, tyrosine and tryptophan biosynthesis	0.77^{b}	0.72^{b}	0.92^{a}	0.93^{a}	0.018	<0.001
Valine, leucine and isoleucine biosynthesis	0.71^{b}	0.67^{b}	0.79^{a}	0.78^{a}	0.011	<0.001
Histidine metabolism	0.58^{b}	0.56^{b}	0.66^{a}	0.66^{a}	0.010	<0.001
Valine, leucine and isoleucine degradation	0.38^{a}	0.32^{a}	0.24^{b}	0.23^{b}	0.015	<0.001
Tyrosine metabolism	0.37^{a}	0.39^{a}	0.3^{b}	0.30^{b}	0.008	<0.001
Tryptophan metabolism	0.27^{a}	0.22^{ab}	0.18^{b}	0.17^{b}	0.010	<0.001
Lysine degradation	0.22^{a}	0.18^{ab}	0.13^{b}	0.13^{b}	0.009	<0.001
Lysine biosynthesis	0.77^{b}	0.76^{b}	0.82^{a}	0.82a	0.007	0.002
Glycine, serine and threonine metabolism	0.82^{ab}	0.8^{b}	0.85^{a}	0.84^{ab}	0.007	0.009
**Carbohydrate Metabolism**						
Butanoate metabolism	0.74^{a}	0.72^{a}	0.63^{b}	0.63^{b}	0.012	<0.001
Propanoate metabolism	0.65^{a}	0.59^{a}	0.5^{b}	0.51^{b}	0.012	<0.001
C5-Branched dibasic acid metabolism	0.29^{b}	0.27^{b}	0.32^{a}	0.32^{a}	0.005	<0.001
Citrate cycle (TCA cycle)	0.66^{ab}	0.64^{b}	0.72^{a}	0.71^{ab}	0.010	0.006
Galactose metabolism	0.63^{b}	0.66^{ab}	0.73^{a}	0.72^{ab}	0.012	0.019
Glycolysis / Gluconeogenesis	1.13^{ab}	1.19^{a}	1.09^{ab}	1.09^{b}	0.013	0.022
Starch and sucrose metabolism	0.88^{b}	0.95^{ab}	0.98^{ab}	0.97^{a}	0.014	0.026
Pentose and glucuronate interconversions	0.43^{b}	0.47^{ab}	0.47^{a}	0.46^{ab}	0.008	0.044
**Energy Metabolism**						
Methane metabolism	1.2^{b}	1.17^{b}	1.31^{a}	1.33^{a}	0.015	<0.001
Oxidative phosphorylation	1.16^{ab}	1.07^{b}	1.26a	1.24a	0.018	<0.001
Carbon fixation pathways in prokaryotes	1.01^{a}	0.98^{a}	1.1^{b}	1.1^{b}	0.011	<0.001
Carbon fixation in photosynthetic organisms	0.59^{b}	0.58^{b}	0.63^{a}	0.63^{a}	0.005	<0.001
Nitrogen metabolism	0.71^{b}	0.71^{b}	0.75^{ab}	0.77^{a}	0.007	0.002
**Enzyme Families**						
Peptidases	1.78^{b}	1.82^{ab}	1.92^{a}	1.92^{a}	0.015	0.002
**Lipid Metabolism**						
Fatty acid metabolism	0.36^{a}	0.31^{a}	0.23^{b}	0.23^{ab}	0.013	<0.001
Fatty acid biosynthesis	0.51^{a}	0.51^{ab}	0.48^{b}	0.48^{ab}	0.004	0.003
Glycerolipid metabolism	0.39^{a}	0.39^{a}	0.37^{ab}	0.36^{b}	0.004	0.004
Biosynthesis of unsaturated fatty acids	0.17^{a}	0.16^{ab}	0.14^{ab}	0.14^{b}	0.005	0.031

**Metabolism of Cofactors and Vitamins**						
Pantothenate and CoA biosynthesis	0.62^{b}	0.59^{b}	0.69^{a}	0.7^{a}	0.009	<0.001
One carbon pool by folate	0.59^{b}	0.59^{b}	0.7^{a}	0.7^{a}	0.010	<0.001
Thiamine metabolism	0.49^{b}	0.48^{b}	0.53^{a}	0.52^{a}	0.005	<0.001
Vitamin B6 metabolism	0.19^{b}	0.17^{b}	0.21^{a}	0.21^{a}	0.004	<0.001
Biotin metabolism	0.15^{ab}	0.14^{b}	0.18^{a}	0.18^{a}	0.004	<0.001
Folate biosynthesis	0.4^{b}	0.39^{b}	0.44^{a}	0.44^{ab}	0.007	0.003
**Replication and Repair**						
DNA replication proteins	1.18^{b}	1.2^{ab}	1.28^{a}	1.29^{a}	0.013	0.002
Mismatch repair	0.81^{b}	0.82^{ab}	0.86^{ab}	0.86^{a}	0.008	0.015
Nucleotide excision repair	0.39^{b}	0.4^{ab}	0.42^{a}	0.42^{ab}	0.004	0.027
**Membrane Transport**						
Transporters	6.25^{ac}	6.55^{a}	5.57^{b}	5.57^{bc}	0.106	<0.001
ABC transporters	3.27^{a}	3.41^{a}	2.83^{b}	2.83^{b}	0.066	<0.001
Phosphotransferase system (PTS)	0.47^{a}	0.6^{a}	0.22^{b}	0.21^{b}	0.034	<0.001
Secretion system	1.49^{a}	1.41^{ab}	1.29^{b}	1.29^{b}	0.028	0.003

Table S2 Affected sub-pathways belonging to the top 10 pathways at KEGG level 2 in the mucosa-associated microbiota. Numbers in the same row with different superscripts indicate a significant difference (*P* < 0.05).

KEGG_Pathways	Jejunum	Ileum	Caecum	Colon	SEM	*P*
**Amino Acid Metabolism**						
Phenylalanine, tyrosine and tryptophan biosynthesis	0.87^{ab}	0.84^{b}	0.89^{ab}	0.91^{a}	0.009	0.017
Tyrosine metabolism	0.34^{ab}	0.36^{a}	0.32^{b}	0.31^{b}	0.008	0.019
Valine, leucine and isoleucine biosynthesis	0.76^{ab}	0.76^{b}	0.79^{a}	0.78^{ab}	0.005	0.03
**Carbohydrate Metabolism**						
C5-Branched dibasic acid metabolism	0.32^{b}	0.32^{b}	0.34^{a}	0.33^{ab}	0.003	0.019
Glycolysis / Gluconeogenesis	1.09^{a}	1.09^{a}	1.03^{b}	1.06^{ab}	0.006	0.011
**Metabolism of Cofactors and Vitamins**						
Biotin metabolism	0.16^{a}	0.16^{a}	0.19^{b}	0.18^{ab}	0.003	0.017
Pantothenate and CoA biosynthesis	0.65^{ab}	0.64^{b}	0.66^{ab}	0.68^{a}	0.005	0.027
**Membrane Transport**						
Phosphotransferase system (PTS)	0.30^{ab}	0.34^{a}	0.26^{b}	0.27^{ab}	0.01	0.028

Table S3 Correlation between the abundance (relative abundance >1% in at least in one location) and significantly affected microbial genera and KEGG pathways of the mucosa-associated microbiota. Only correlation coefficients with *P* value <0.05 and |r|>0.5 are presented.

Genus	KEGG pathway	KO numbers	Correlation coefficient	P Value
Lactobacillus	Glycolysis / Gluconeogenesis	ko00010	0.71	<0.001
Lactobacillus	Valine, leucine and isoleucine biosynthesis	ko00290	-0.65	<0.001
Lactobacillus	C5-Branched dibasic acid metabolism	ko00660	-0.63	<0.001
Lactobacillus	Phosphotransferase system (PTS)	ko02060	0.59	<0.001
Streptococcus	Glycolysis / Gluconeogenesis	ko00010	0.73	<0.001
Streptococcus	Phosphotransferase system (PTS)	ko02060	0.61	<0.001
Streptococcus	Valine, leucine and isoleucine biosynthesis	ko00290	-0.52	0.001
Sphingomonas	Tyrosine metabolism	ko00350	0.78	<0.001
Sphingomonas	Pantothenate and CoA biosynthesis	ko00770	-0.58	<0.001
Sphingomonas	Phenylalanine, tyrosine and tryptophan biosynthesis	ko00400	-0.52	<0.001
Sphingomonas	Valine, leucine and isoleucine biosynthesis	ko00290	0.51	<0.001
Campylobacter	Glycolysis / Gluconeogenesis	ko00010	-0.62	<0.001
Campylobacter	Biotin metabolism	ko00780	0.61	<0.001
Helicobacter	Biotin metabolism	ko00780	0.64	<0.001
